# Patient-specific instrumentation improved three-dimensional accuracy in total knee arthroplasty: a comparative radiographic analysis of 1257 total knee arthroplasties

**DOI:** 10.1186/s13018-019-1465-6

**Published:** 2019-12-12

**Authors:** Leo Pauzenberger, Martin Munz, Georg Brandl, Julia K. Frank, Philipp R. Heuberer, Brenda Laky, Eva Schwameis, Werner Anderl

**Affiliations:** 1 Vienna Shoulder & Sports Clinic, Vienna, Austria; 2 Sports Surgery Clinic, Dublin, Ireland; 3 Medical University of Vienna, Vienna, Austria; 4 Austrian Research Group for Regenerative and Orthopedic Medicine (AURROM), Vienna, Austria; 5 Health Pi, Vienna, Austria; 6 MedSciCare, Vienna, Austria; 7 Shoulder & Sports Center, Mödling, Austria

**Keywords:** Total knee arthroplasty, Patient-specific instrumentation, Outliers, Three-dimensional component position, Accuracy

## Abstract

**Background:**

The purpose of this study was to compare restoration of mechanical limb alignment and three-dimensional component-positioning between conventional and patient-specific instrumentation in total knee arthroplasty.

**Methods:**

Radiographic data of patients undergoing mobile-bearing total knee arthroplasty (*n* = 1257), using either conventional (*n* = 442) or patient-specific instrumentation (*n* = 812), were analyzed. To evaluate accuracy of axis restoration and 3D-component-positioning between conventional and patient-specific instrumentation, absolute deviations from the targeted neutral mechanical limb alignment and planned implant positions were determined. Measurements were performed on standardized coronal long-leg and sagittal knee radiographs. CT-scans were evaluated for accuracy of axial femoral implant rotation. Outliers were defined as deviations from the targeted neutral mechanical axis of > ± 3° or from the intraoperative component-positioning goals of > ± 2°. Deviations greater than ± 5° from set targets were considered to be severe outliers.

**Results:**

Deviations from a neutral mechanical axis (conventional instrumentation: 2.3°± 1.7° vs. patient-specific instrumentation: 1.7°± 1.2°; *p* < 0.001) and numbers of outliers (conventional instrumentation: 25.8% vs. patient-specific instrumentation: 10.1%; *p* < 0.001) were significantly lower in the patient-specific instrumentation group. Significantly lower mean deviations and less outliers were detected regarding 3D-component-positioning in the patient-specific instrumentation compared to the conventional instrumentation group (all *p* < 0.05).

**Conclusions:**

Patient-specific instrumentation prevented from severe limb malalignment and component-positioning outliers (> ± 5° deviation). Use of patient-specific instrumentation proved to be superior to conventional instrumentation in achieving more accurate limb alignment and 3D-component positioning, particularly regarding femoral component rotation. Furthermore, the use of patient-specific instrumentation successfully prevented severe (> 5° deviation) outliers.

## Background

Restoration of neutral mechanical limb alignment and exact component positioning have been reported to be essential for satisfactory long-term outcome after total knee arthroplasty (TKA) [1, 2]. Various studies showed that coronal limb alignment is an important factor in implant durability, as outliers in the frontal plane had a significantly higher risk for early loosening and polyethylene wear with decreased overall implant survival [1, 2]. Mechanical malalignment and component malpositioning have also been implicated as causative for unsatisfactory clinical outcome [3–5]. Although the clinical importance of a neutral mechanical alignment on implant longevity has recently become a matter of discussion, there is currently no better parameter to aim for when performing TKA [1, 5, 6].

Despite correct surgical techniques, expanding knowledge and experience with manual instrumentation systems, imprecise alignment, and component positioning remain common issues in conventional TKA [7]. Consequentially, various efforts have been made to introduce technology to aid the surgeon in reliably improving accuracy of implantation in TKA over the last years with computer-assisted surgery leading the way [8]. Although computer-assisted surgery could improve surgical accuracy, it came with the disadvantages of complex instrumentation and longer surgery times.

As a contemporary alternative, patient-specific instrumentation (PSI) has been introduced to joint reconstruction in recent years. In short, for the purpose of PSI, computed tomography (CT) scans or magnetic resonance images (MRI) are used for preoperative three-dimensional (3D) planning and subsequent production of patient-specific cutting guides providing a unique fit on femur and tibia for exact bone resection and component positioning.

As the potential benefits of these systems, such as reduced surgical time and superior accuracy of knee replacement, come at the cost of increased economic and logistic expenses, PSI came under scrutiny quickly [9–11]. Although expectations were high, there is no clear consensus in literature regarding accuracy, reliability, and actual value of PSI in knee replacement surgery [12–15].

Therefore, the purpose of the present study was to compare radiological limb alignment and 3D-component positioning between conventional instrumentation (CVI) and a CT-based PSI system in primary, mobile-bearing TKA in the setting of a single-center over a period of multiple years. We hypothesized that PSI would be superior regarding mechanical alignment restoration and 3D-component positioning compared with CVI.

## Methods

The current study had Institutional Review Board approval (#EK201305). The present retrospective cohort study (Levels of Evidence III) was based on analyses of prospectively collected radiographic data of patients, who underwent surgery with a mobile-bearing TKA system (GMK® Primary, Medacta International S.A., Castel San Pietro, Switzerland) regardless of preoperative varus or valgus deformity in a single center during the period 2007 to 2013. From 2007 to 2010, the GMK Primary system was only available with conventional instrumentation, whereas the MyKnee PSI technology was available starting from 2010. Since then, the included conventionally performed TKA group consisted of patients, who were scheduled on short notice without a minimum lead time of 2 weeks to procure the PSI and refused to wait for a later surgical date, refused performance of a preoperative CT, or opposed surgery with by then fairly new PSI technology.

Of initially available 1315 postoperative radiographs, 58 cases were excluded due to previous hip arthroplasty (*n* = 33), previous fractures of the femur or tibia (*n* = 5), implants around the knee (*n* = 7), or insufficient quality of radiographs (*n* = 13). Complete radiographic data of 1257 knee arthroplasties were evaluated including 442 cases performed with CVI and 815 cases with PSI. Additionally, 138 (CVI: *n* = 44, PSI: *n* = 94) available postoperative CT-scans were analyzed.

Preoperative CT-scans including sections of the femoral head, knee, and ankle, with the patient supine and the leg in complete extension according to a standardized protocol (MyKnee®, Medacta International S.A., Castel San Pietro, Switzerland), were made prior to surgery. Generated images were then uploaded to the company website for further processing. 3D-bone-models of the knee and cutting blocks were created and planned by an engineer according to the preferences of the surgeons. The planning targets for all patients were a neutral mechanical axis, a physiological joint line, a tibial slope between 0° and 6° to restore an anatomic situation, a flush fit of the femoral component on the anterior cortex to avoid notching (0° to 4° flexion), a femoral component rotation parallel to the transepicondylar axis, and a tibial component rotation oriented according to the tibial tuberosity. These intraoperative goals were the same for the CVI group, whereas planning of lower limb axis, coronal, and sagittal component position was done manually on standard long-leg and knee radiographs (mediCAD, Hectec GmbH, Altdorf, Germany).

All included patients underwent implantation using cemented mobile-bearing total knee prosthesis (GMK® Primary, Medacta International S.A., Castel San Pietro, Switzerland) without patellar resurfacing. After a midline skin incision, a medial or, in case of more than 5° valgus alignment, lateral parapatellar arthrotomy was performed. In the CVI group, the standard GMK® instrumentation system including an extra-medullary guidance rod for tibial- and an intra-medullary guidance rod for femoral alignment was used. For use of PSI, the tibial and femoral footprint areas were carefully cleaned of the remaining cartilage using the electro-cautery for an exact fit of the cutting blocks, and after pinning, bone cuts were performed in accordance to the preoperative planning. Suggested resections and femoral rotation were checked for accordance to the preoperative plan. Although the PSI system provided the possibility for intraoperative control, tibial component rotation was determined manually with the goal of orienting the tibial plateau according to the medial third of the tibial tuberosity. Therefore, an analysis of tibial component positioning was not included in the present study. Necessary removal of osteophytes and soft tissue balancing were carefully performed in both groups. All surgeries were performed by three senior surgeons trained in total joint reconstruction, whereas all operated on patients with CVI as well as with PSI. Mobilization and physiotherapy started on the first postoperative day in both groups. Continuous passive motion was used complementarily until a minimum flexion of 90° was possible.

Standardized coronal long-leg, sagittal knee joint, and tangential patella radiographs, which were taken prior to surgery and on the day of hospital discharge, were analyzed. CT-scans were used to compare femoral component rotation (FCR) of patients receiving PSI with the standard technique.

**Fig. 1:**
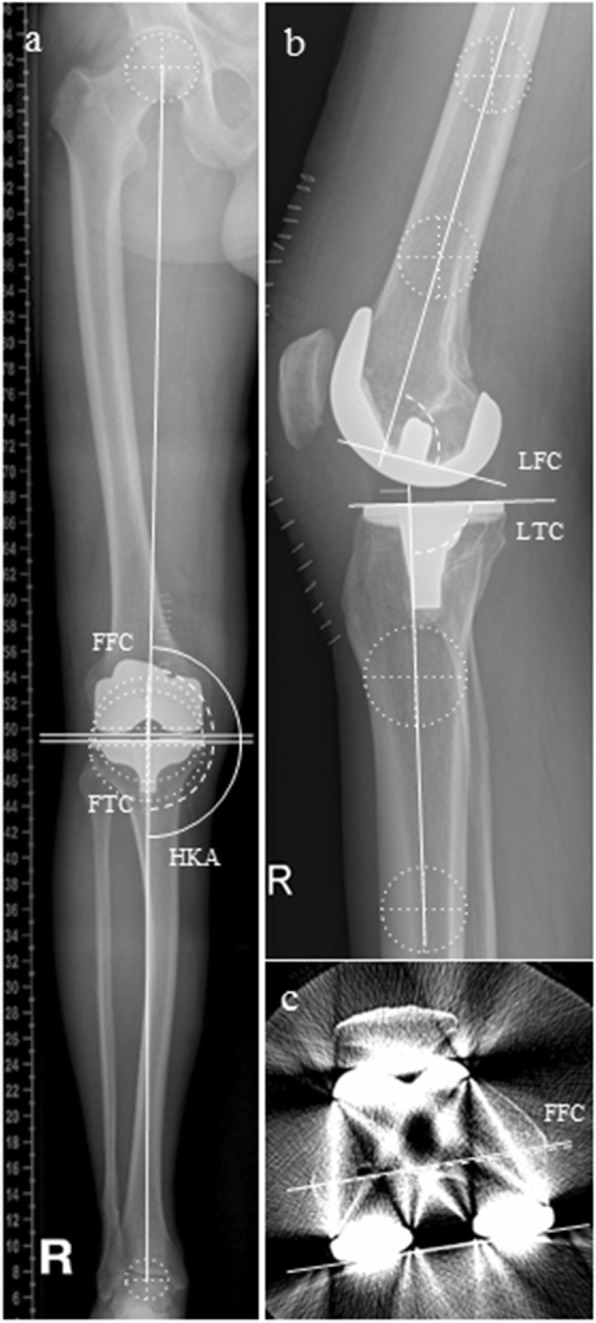
Postoperative radiographs (**a**, **b**) and CT-scan (**c**) of a right leg and knee, depicting the methods of angle measurements. The hip-knee-ankle angle (HKA), frontal femoral (FFC), and frontal tibial (FTC) component angle were measured on long-leg weight-bearing radiographs (**a**). Lateral femoral (LFC) and lateral tibial (LTC) component angles were evaluated on sagittal short view radiographs (**b**). Axial femoral component rotation (FCR) was analyzed on postoperative CT-scans of the knee (**c**)

To evaluate overall limb alignment, the hip-knee-ankle angle (HKA) was measured on weight-bearing long-leg radiographs, whereas an HKA of over 180° was defined as valgus and a HKA under 180° was defined as varus alignment. Frontal femoral component (FFC) position was defined as the angle between the femoral mechanical axis and the line formed by the distal femoral condyles. Frontal tibial component (FTC) position was measured as the angle between the mechanical axis and the tibial plateau. For both measurements, an angle above or below 90° was considered a valgus or varus position. Lateral femoral (LFC) and tibial (LTC) component positions were defined as the angle between the femoral or tibial axis and the respective implant surfaces. FCR was assessed on axial CT scans by measuring the angle between the posterior component margins and the transepicondylar line (Fig. 1).

Radiographic measurements were performed by two independent examiners, who were not involved in the surgical procedures and blinded regarding the used instrumentation. A random subset of 30 patients of each instrumentation group was measured twice by each examiner at two different time points to assess inter- and intra-rater reliability using interclass correlation coefficient (ICC). This analysis revealed intra- and inter-rater measurement agreement for all examined parameters (HKA, FFC, FTC, LFC, LTC, and FCR) ranging from 0.932 to 0.996 and from 0.931 to 0.996, respectively.

To analyze accuracy of mechanical axis restoration and 3D-component positioning between the CVI and PSI group, deviations from the targeted neutral mechanical limb alignment and 3D-component positioning in degrees were calculated. Outliers were defined as deviations from the targeted neutral mechanical axis of more than ± 3° (HKA) or from the intra-operative component positioning goals of more than ± 2° (FFC, FTC, LFC, LTC, and FCR). Deviations greater than ± 5° from set targets were considered to be severe outliers [16].

Descriptive statistic was used to present patients demographics. Distribution of data was assessed by a visual inspection of histograms and the Kolmogorov–Smirnov test. The independent or paired *t* test for normally distributed variables or the nonparametric Mann–Whitney *U* test or Wilcoxon signed rank test was performed to compare continuous variables. Fisher’s exact or *Χ*^2^ test were used to analyze categorical variables. With the current sample size, there was a 95% power to detect an effect size of > 0.21 for the deviation from a targeted neutral mechanical axis. Statistical significance was reported at a *p* value of < 0.05 level (two-sided). All statistical analyses were performed in SPSS21® (IBM® Corporation, Armonk, USA).

## Results

Baseline patient characteristics were comparable between the instrumentation groups with significant differences only in the ratio of female to male patients and distribution of preoperative osteoarthritic degeneration according to the Kellgren–Lawrence classification (Table 1).

**Table 1:** Study group characteristics

	CVI group (*n* = 442)	PSI group (*n* = 815)	*p* value
Age (years)	69.2 (9.5)	68.9 (8.5)	0.515
Gender			
Female	70.4% (311)	62.2% (507)	0.004
Male	29.6% (131)	37.8% (308)	
Surgical side			
Left	47.3% (209)	47.7% (389)	0.906
Right	52.7% (233)	52.3% (426)	
Kellgren–Lawrence classification			
2°	36% (159)	18.2% (149)	< 0.001
3°	53.8% (238)	44.5% (363)	
4°	10.2% (45)	37.3% (304)	
Preoperative HKA	176.7° (7.8)	176.6° (9.0)	0.905
Preoperative limb alignment			
Varus	71.0% (314)	70.6% (575)	0.784
Valgus	26.5% (117)	27.5% (224)	
Neutral	2.5% (11)	2.0% (16)	

Values are given as mean and standard deviation in parentheses or proportion and number of cases in parentheses, wherever appropriate

*CVI* conventional instrumentation, *HKA* hip-knee-ankle angle, *PSI* patient-specific instrumentation

Significant HKA improvements from pre- to postoperative were detected in both groups (*p* < 0.001; Tables 1 and 2). Analysis of mean limb alignment and component positioning showed statistically significant differences (with the exception of LTC) between the two groups (Table 2).

**Table 2:** Postoperative measurements

	CVI group (*n* = 442)	PSI group (*n* = 815)	*p* value
HKA	180.0° (2.9)	179.5° (2.0)	0.001
FFC	90.8° (2.1)	90.0° (1.6)	< 0.001
FTC	89.2° (1.8)	89.4° (1.5)	0.033
LFC	87.5° (2.9)	86.5° (2.2)	< 0.001
LTC	85.6° (2.8)	85.6° (2.4)	0.728
FCR^a^	2.4° (1.6)	1.1° (0.6)	< 0.001

Values are given as mean and standard deviation in parentheses

*CVI* conventional instrumentation, *FCR* femoral component rotation, *FFC* frontal femoral component angle, *FTC* frontal tibial component angle, *HKA* hip-knee-ankle angle, *LFC* lateral femoral component angle, *LTC* lateral tibial component angle, *PSI* patient-specific instrumentation

^a^CT scans for the evaluation of rotational component alignment were available for 44 cases of the CVI group, and for 94 case of the PSI group.

**Fig. 2:**
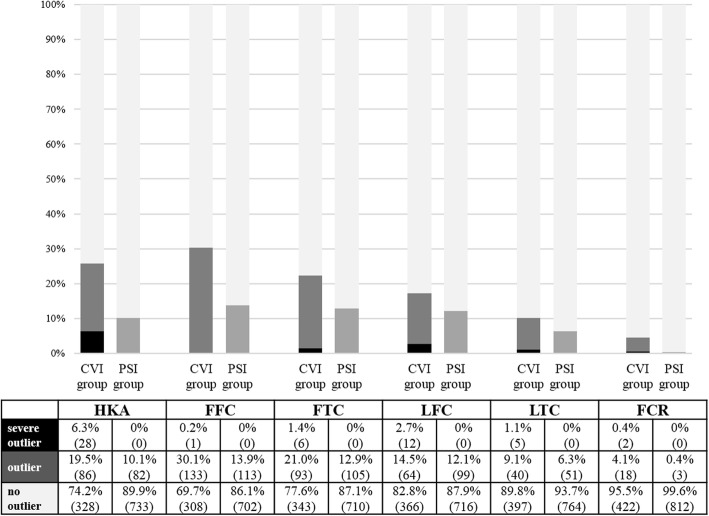
Accuracy of mechanical axis restoration and 3D-component positioning of the conventional instrumentation (CVI) and the patient-specific instrumentation (PSI) group. Outliers (dark grey bars) were defined as deviations from the targeted neutral mechanical axis of more than ± 3° (hip-knee-ankle angle, HKA) or from the intra-operative component positioning goals of more than ± 2° (frontal femoral component angle, FFC; frontal tibial component angle, FTC; lateral femoral component angle, LFC; lateral tibial component angle, LTC; femoral component rotation, FCR; dark grey bars). Deviations greater than ± 5° from set targets were considered to be severe outliers (black bars). CT scans for the evaluation of rotational component alignment were available for 44 cases of the CVI group, and for 94 case of the PSI group. Values are presented as percentages and numbers of cases in parentheses

Significantly less patients in the PSI compared to the CVI group experienced postoperative outliers regarding HKA (10.1% vs. 25.8%, *p* < 0.001), FFC (13.9% vs. 30.3%, *p* < 0.001), FTC (12.9% vs. 22.4%, *p* < 0.001), LFC (12.2% vs. 17.2%, *p* = 0.017), LTC (6.3% vs. 10.2%, *p* = 0.014), and FCR (3.2% vs. 39.2%, *p* < 0.001). Furthermore, there were significantly more severe outliers detected in the CVI compared to the PSI group regarding HKA (0% vs. 6.3%, *p* < 0.001), FTC (0% vs. 1.4%, *p* = 0.002), LFC (0% vs. 2.7%, *p* < 0.001), and LTC (0% vs. 1.1%, *p* = 0.005). Only 0.2% FFC and 3.9% FCR were found to be severe outliers in the CVI group, which was not significantly different to the PSI group. The numbers of all outliers are presented in Fig. 2.

PSI also significantly reduced the mean absolute deviation from the targeted neutral mechanical alignment and planned component position in all planes (Table 3).

**Table 3:** Mean absolute deviation from target

	CVI group (*n* = 442)	PSI group (*n* = 815)	*p* value
HKA	2.3° (1.7)	1.7° (1.2)	< 0.001
FFC	1.8° (1.3)	1.3° (0.9)	< 0.001
FTC	1.5° (1.2)	1.3° (0.9)	< 0.001
LFC	0.9° (1.4)	0.7° (1.0)	0.011
LTC	0.6° (1.2)	0.4° (0.9)	0.003
FCR^a^	2.5° (1.4)	1.1° (0.6)	< 0.001

Values are given as mean and standard deviation in parentheses

*CVI* conventional instrumentation, *FCR* femoral component rotation, *FFC* frontal femoral component angle, *FTC* frontal tibial component angle, *HKA* hip-knee-ankle angle, *LFC* lateral femoral component angle, *LTC* lateral tibial component angle, *PSI* patient-specific instrumentation

^a^CT scans for the evaluation of rotational component alignment were available for 44 cases of the CVI group, and for 94 case of the PSI group

## Discussion

This comparative study between conventional and patient-specific instrumentation in TKA presents results of a high number of cases (*n* = 1257) including standardized radiographic evaluation and CT scan data. The most important findings of the present study were that CT-based PSI significantly reduced the number of limb alignment (> 3°) and component positioning outliers (> 2°) in all planes, while protecting against the risk of severe outliers (> 5°). Furthermore, significantly more accurate mechanical alignment restoration and 3D-component positioning could be detected in the PSI compared to the CVI group.

The impetus for development of PSI was to aid the surgeon in achieving reproducible accuracy in TKA to eventually improve outcome and implant longevity. Considerable deviations from a targeted neutral mechanical axis have traditionally been implicated as cause for inferior clinical outcome and decreased implant survival [1, 2, 4, 5, 17, 18]. Although this longstanding concept has been challenged recently [19–22], it is today’s gold standard for intraoperative limb alignment. Less literature is available on the impact of individual component positioning on clinical outcome and implant survival. Incorrect tibiofemoral coronal implant placement has been implicated as reason for revision surgery [2]. Specifically, if an overall malalignment is based on certain biomechanically disadvantageous component position combinations, long-term failure rates have been shown to dramatically increase to over 10%, with an up to 54 times higher risk for failure than correctly placed implants [2]. Recent clinical studies showed that there is no relevant correlation between posterior tibial slope and kinematics after TKA [23–25]. However, tibial slopes below 0° and over 7° have been associated with increased risk of failure [25]. Inadequate femoral component rotation can negatively affect patellar tracking and knee kinematics [26]. An isolated internal rotation of the femoral component has been associated with anterior knee pain, instability, stiffness, and early revision [27–31].

Various meta-analyses comparing the number of outliers (> 3°) from a neutral mechanical axis of PSI and CVI found results between the instrumentation techniques to be comparable at best [12, 13, 15]. Similar disappointing outcomes have been reported for multi-planar component positioning [12, 13, 15]. Contrary to these inconsistent results, the present study suggests that PSI, if used deliberately, has the potential to significantly increase precision of intended 3D-component positioning and mechanical axis restoration, while at the same time significantly reducing the number of outliers in all planes.

Reasons for the often inconsistent results with PSI may originate in part in the different used PSI systems, nature of performed studies, varying surgical techniques, and disparity of measurements: There is considerable heterogeneity in accuracy between the available PSI systems. For example, one recent meta-analysis [13] found no difference in the overall number of HKA outliers between CVI (22.7%) and PSI (23.2%), whereas a subgroup analysis showed that there were substantial differences between various PSI systems. While three systems (Visionaire, Smith and Nephew, Memphis, TN; PSI, Zimmer, Warsaw, IN; TruMatch, DePuy, Warsaw, IN) provided comparable rates of outliers to CVI, one PSI system (Signature®, Biomet, Warsaw, IN) actually showed a 54% increased risk of producing an HKA outlier.

Another reason for inconsistency with PSI may originate in part from the nature of performed studies beyond issues of study design, PSI systems, varying surgical techniques, and disparity of measurements. Although being formally of high quality, studies might lack the sample size or length of study period necessary to account for learning curves for implementing needed logistics, optimizing the preoperative planning process, and adapting the surgical strategy for successful implementation of PSI.

In contrast to the variable results for limb alignment restoration and component positioning in the coronal and sagittal plane, there is predominant consensus about the superiority of PSI to achieve accurate femoral implant rotation with simultaneous reduction of outliers [12, 13, 15, 16, 29, 32]. Our results agree with the literature, proving femoral component rotation as benefiting the most from the use of PSI. Not only was there a significant improvement in accuracy (1.4° less deviation from target per case) but also a distinct reduction of outliers in the PSI group (CVI: 39.2% vs. PSI: 3.2%).

Ultimately, PSI has to be treated as a tool to aid the surgeon in potentially improving accuracy of TKA. Although promising, PSI is still in a relatively early phase of development and cannot yet completely mitigate against all pitfalls of TKA surgery. It is absolutely necessary for surgeons to be actively involved in the preoperative planning process [33–37]. Intraoperative PSI-specific details have to be considered and surgical techniques adjusted where necessary. Double-checking alignment and bony resections at relevant surgical steps according to traditional concepts is mandatory, because, for now, successful outcome of TKA is still the responsibility of the performing surgeon.

The present study has certain limitations. The results pertain only to the used MyKnee® PSI system and, thus, generalization of results to other PSI systems should be done with caution. The number of available CT-scans for axial component analysis was limited. In order to minimize costs and extensive radiation exposure, no additional CT scans were performed for the purpose of this study. Surgeries were performed by three different surgeons. Although this adds variability, it reflects clinical reality of multiple surgeons performing TKA at an institution. The number of knees with severest osteoarthritic changes was higher in the PSI group. However, if anything, this underlines the ability of PSI to reliably aid surgeons in achieving their desired radiological results independent of disease severity. Lastly, the present study was a radiographic evaluation in order to assess the accuracy of PSI compared to CVI. Future investigations are needed for the assessment of according clinical outcome.

## Conclusions

In the current comparative study, we investigated the accuracy of conventional and patient-specific instrumentation in TKA (*n* = 1257) using standardized radiographic evaluation and CT scan data. The used CT-based PSI system significantly reduced the number of limb alignment (> 3°) and component positioning outliers (> 2°) in all planes, while protecting against the risk of severe outliers (> 5°). Furthermore, significantly more accurate restoration of mechanical alignment and 3D-component positioning could be achieved in the PSI group.

## Data Availability

All data generated or analysed during this study are included in this published article.

## Abbreviations

3D: Three-dimensional
CT: Computed tomography
CVI: Conventional instrumentation
FCR: Femoral component rotation
FFC: Frontal femoral component
FTC: Frontal tibial component
HKA: Hip-knee-ankle angle
LFC: Lateral femoral component
LTC: Lateral tibial component
MRI: Magnetic resonance images
PSI: Patient-specific instrumentation
TKA: Total knee arthroplasty
